# Lymph node enlargement after definitive chemoradiotherapy for clinical stage I esophageal squamous cell carcinoma

**DOI:** 10.1186/1471-2407-14-706

**Published:** 2014-09-24

**Authors:** Yoshito Hayashi, Tsutomu Nishida, Masahiko Tsujii, Shusaku Tsutsui, Katsumi Yamamoto, Fumiaki Isohashi, Makoto Yamasaki, Hiroshi Miyata, Motohiko Kato, Takuya Yamada, Shinichiro Shinzaki, Hideki Iijima, Kazuhiko Ogawa, Yuichiro Doki, Tetsuo Takehara

**Affiliations:** Department of Gastroenterology and Hepatology, Osaka University Graduate School of Medicine, 2-2 Yamadaoka, Suita, Osaka, 565-0871 Japan; Department of Radiation Oncology, Osaka University Graduate School of Medicine, Suita, Osaka, Japan; Department of Gastroenterological Surgery, Osaka University Graduate School of Medicine, Suita, Osaka, Japan

**Keywords:** Esophageal carcinoma, Chemoradiotherapy, Lymph node enlargement

## Abstract

**Background:**

Chemoradiotherapy (CRT) is an effective modality for stage I esophageal squamous cell carcinoma (ESCC). However, salvage treatments are often required even if complete response (CR) has been achieved. To this end, it is important to accurately diagnose lymph node or other organ metastatic recurrences. Note that lymph node enlargements (except metastatic recurrence) are often detected during the follow-up period after CRT. The purpose of this study was to elucidate the clinical characteristics of lymph node enlargement after CRT.

**Methods:**

In this retrospective cohort study, patients diagnosed with stage I (T1 [submucosal invasion] N0M0) ESCC were treated with cisplatin and 5-fluorouracil concurrently with radiotherapy. A total of 55 patients were enrolled in the study from February 2006 to August 2011.

**Results:**

The median follow-up period was 46 months. The 3-year overall and progression-free survival rates were 90.7% and 71.2%, respectively, and the CR rate was 87.2% (48/55). Nine of the 48 CR patients were finally diagnosed with recurrences, including 7 lymph node metastases and 2 local recurrences. Lymph node enlargement was initially identified in 20 of the total 55 patients during the follow-up; 9 patients were finally diagnosed with lymph node recurrence, whereas 11 patients had benign reactive lymph node enlargement.

**Conclusion:**

The present study demonstrated the high incidence of enlarged lymph nodes after CRT for stage I ESCC. It is important to accurately distinguish between benign lymph node enlargement and recurrent lymph nodes to avoid unnecessary salvage treatments.

## Background

Esophageal carcinoma is the major cause of cancer-related mortality in the world and also in Japan [1]. Surgical esophagectomy with lymphadenectomy is considered to be the standard treatment for patients in clinical stages I to III ESCC [2, 3]. Recent improvements of endoscopic techniques (non-surgical treatments) have allowed organ preservation in patients with mucosal ESCC. However, endoscopic resection (ER) is not indicated for ESCC with submucosal invasion. Moreover, there is a high incidence of lymph node occult metastasis in 10% to 30% of the patients with submucosal cancer. Today, these patients are usually treated surgically. The survival rate of patients with submucosal tumors treated surgically at 3 years is > 80%, but esophagectomy is an invasive procedure and has the risk of postoperative morbidity. For the patients in stages II and III ESCC, postoperative chemotherapy is superior to surgery alone in disease free survival and preoperative chemotherapy leads to the superiority to postoperative chemotherapy in overall survival [4, 5], which suggests that chemotherapy is efficacious in suppressing lymph node recurrences. Several reports have recently confirmed that concurrent chemoradiotherapy (CRT) is a more effective treatment in patients with advanced ESCC, compared to radiotherapy alone [6–8]. CRT is considered to be less invasive because of the better quality of life after the treatment compared to esophagectomy. Definitive CRT is considered to have an indication not only for the advanced stage but for the early stage as well [9–11]. A phase II study has demonstrated that definitive CRT is a favorable alternative to esophagectomy in patients with clinical stage I ESCC [10, 12].

CRT seems to be an effective modality, particularly for submucosal ESCC without lymph node metastasis. However, it is known that patients with submucosal invasion have invisible lymph node metastases at the time of diagnosis. Accordingly, they are considered to be at high risk for lymph node recurrence after CRT, and salvage treatments are often required to improve their survival, although they had achieved complete response (CR). To detect metastatic or recurrent lesions as soon as possible, endoscopy and thoraco-abdominal computed tomography (CT) are regularly performed for monitoring after CRT. If a recurrence occurred locally in the mucosal layer, it is easy to diagnose with endoscopic biopsy and ER is a useful salvage modality to control in such cases. On the contrary, it is difficult to accurately diagnose lymph node recurrence after CRT because little is known about the recurrence pattern after CRT for patients with clinical stage I ESCC. We experienced several cases in which lymph node enlargements were detected after CRT. Although surgical esophagectomy was performed in the early period, we observed that a few cases were pathologically benign, which suggested that benign lymph node enlargement that was reactive to the treatment might mimic metastasis in certain cases. The aim of this study was to elucidate the clinical characteristics of lymph node enlargement after CRT.

## Methods

### Patient population

This retrospective study used the database at the Department of Gastroenterology and Hepatology of Osaka University Hospital. From February 2006 to August 2011, 55 consecutive patients with stage I (T1 [submucosal cancer] N0 M0) ESCC who were treated with CRT were analysed. TNM staging was determined according to the Union for International Cancer Control criteria. None of the patients chose the surgical treatment. The median patient age was 66 years (range, 49–82 years). The clinical stage was diagnosed by endoscopy, endoscopic ultrasonography, cervical and thoraco-abdominal CT. Tumor localization was identified by combining chromoendoscopy with Lugol staining. Tumor invasion depth was evaluated using magnification endoscopy with narrow band imaging (NBI) and endoscopic ultrasonography in addition to conventional CT. The CRT was performed as following criteria: 1) Eastern Cooperative Oncology Group performance status of 0–2; 2) leucocyte count >3,000/m^3^, platelet count >100,000/m^3^; 3) aspartate aminotransferase or alanine transferase level within 3 times the normal upper limit; 4) creatinine level <1.5 mg/dl and creatinine clearance >50 ml/min; and 5) no other serious complications. All patients had adequate hepatic and renal functions and performance status scores of 0. The tumor histological type was diagnosed as squamous cell carcinoma based on an endoscopic biopsy. This study was approved by the Institutional Review Board of Osaka University.

### Chemoradiotherapy

All patients were treated with cisplatin and 5-FU chemotherapy. Cisplatin was administered at a dose of 70 mg/m^2^ body surface area on Day 1 and Day 29, and 5-FU was administered at a dose of 700 mg/m^2^ per day by continuous infusion for 24 hours on Days 1–5 and Days 29–33. Nedaplatin was administered instead of cisplatin to 3 patients on Day 29 because of renal dysfunction induced by cisplatin. The concurrent radiotherapy consisted of external administrations of 2 Gy daily to a total dose of 60 Gy without a planned break. The gross tumor volume was limited to the primary tumor. The planning target volume was defined by adding 2- to 3-cm margins above and below the tumor without prophylactic lymph node coverage according to the clips marked during the endoscopic procedure. The lateral, anterior, and posterior margins were limited to 1–2 cm. The radiation therapy field comprised the planning target volume for up to 40 Gy with anterior/posterior opposed portals and exposed to an additional 20 Gy with bilateral oblique portals excluding the spinal cord.

### Assessments of response, recurrence and toxicity after CRT

After CRT, the clinical response was assessed with endoscopic observation accompanied by biopsy specimens and a thoraco-abdominal multidetector CT scan. CR was defined when a tumor was not detected by endoscopic observation and CT scan for >4 weeks. First, the initial evaluation was performed 1 month after CRT and subsequently followed up at 3-month intervals up to 1 year and subsequently at 6 months and up to 5 years by endoscopy and thoraco-abdominal CT scan or until recurrence was diagnosed. Metachronous esophageal recurrence out of the radiation field was not included in the present study. A lymph node enlargement was defined when the size of a lymph node was larger than the initial size before CRT or newly detected by CT scan. The early cases were diagnosed as a recurrence or a benign enlargement pathologically by surgical resection. In the later cases, the lymph node enlargement was followed by CT scan with closed interval. During the closed follow-up, the enlarged lymph nodes were diagnosed as a recurrence when they increased in size and number in the CT scan. When the exacerbation was not detected for more than 6 months, lymph node enlargement was defined as “benign”.

Adverse events were retrospectively evaluated using the Common Terminology Criteria version 4.0. We evaluated therapeutic late toxicity according to the Radiation Therapy Oncology Group/European Organization for Research and Treatment of Cancer late radiation morbidity scoring schema (available at http://www.rtog.org/).

### Statistical analysis

If there were missing cases, the date of the last observation was defined as the censor date. The overall survival was calculated using the Kaplan-Meier method from the date of CRT initiation to death from any cause. Cumulative progression-free survival was calculated using the Kaplan-Meier method from the date of CRT initiation to the recurrence or death from any cause. A Wilcoxon test was performed to compare diameters and intervals until lymph node enlargement between benign enlargement and recurrence. All analyses were performed using JMP software (V.10.0.2, SAS Institute Inc., Cary, NC, USA).

## Results

### Patient characteristics

The median follow-up period after initiating CRT was 46 months (range, 2–83 months). The main clinical characteristics of the patients are presented in Table 1. Before the treatment, 5 patients had been treated for malignancies in other organs, which were inactive when CRT was performed for ESCC. During the follow-up period, the second primary cancers in the other organs were observed in 10 patients: pancreas, skin, and prostate, 1; biliary duct, 1; urinary tract, 1; pharynx, 1; colon, 1; lung and stomach, 1; vocal cord and stomach, 1; valve, 1; thyroid, 1; and stomach, 1. Esophageal intraepithelial neoplasia, which occurred metachronously out of the radiation therapy field, was detected in 3 patients after CRT; all of whom were treated with ER.

**Table 1:** **Patient characteristics**

Number of patients	55
Gender male/female	49/6
Age, median [range] (yrs)	66 [49–82]
Performance status, 0/1/2	55/0/0
Tumor main location	
Upper thoracic	7
Middle thoracic	34
Lower thoracic	14
Inactive multiple cancers in other organ	
no/yes	50/5
Median follow-up period [range] (months)	46 [2–83]

### Adherence and adverse events

All but 2 patients successfully underwent total treatment, and the treatment completion rate was 96%. One of the 2 patients received only 52 Gy irradiation because he withdrew further treatment, and the other patient received an 80% dose of chemotherapy on Days 29–33 because of digestive toxicity. One patient died 2 weeks after CRT completion. The causal correlation with the treatment was not confirmed; however, treatment-related death was suspected.

With regard to hematologic adverse events, grade 3 or 4 leukocytopenia was observed in 8 patients, and grade 3 anaemia was observed in 1 patient. Grade 3 appetite loss and nausea were seen in 3 patients, and grade 3 mucositis was observed in 3 patients. Late toxicity events were observed 3 to 24 months after the treatment. Grade 3 interstitial lung disease was observed in 1 patient (1.8%). Grade 3 pericardial effusions were observed in 3 patients (5.5%). Two of these patients had been treated for cardiac disease before CRT.

### Treatment efficacy

**Figure 1:**
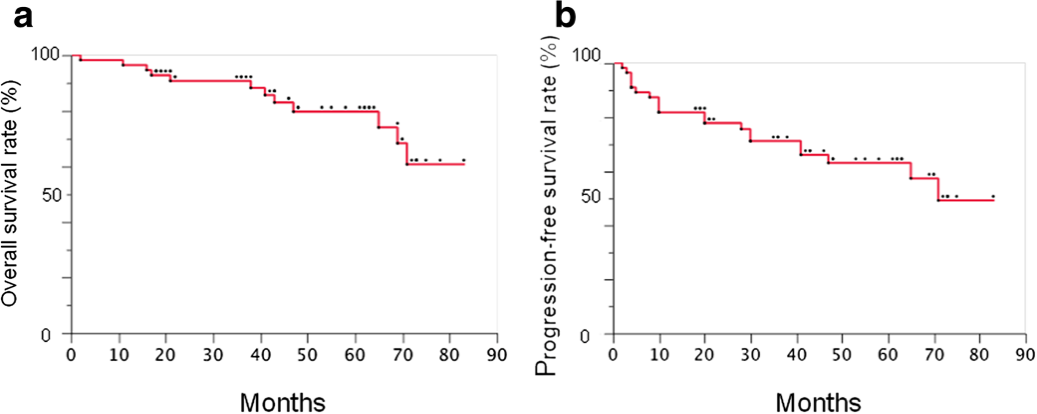
**Treatment Efficacy of CRT. a**. Cumulative overall survival curve. The 3-year overall survival rate was 90.7%. **b**. Cumulative progression-free survival curve. The 3-year progression-free survival rate was 71.2%.

The 3-year overall survival rate in all patients was 90.7% (Figure 1a). The CR rate was 87.2% (48/55). Although 6 non-CR patients, except for 1 patient who was suspected of treatment-related death, underwent salvage treatment, including surgery and chemotherapy, 2 died because of lymph node recurrence and liver metastasis (Table 2). Of the 48 CR patients, 5 died from lymph node recurrences, and 4 patients died without ESCC recurrences during the follow-up period: urinary tract cancer, 1; biliary tract cancer, 1; sudden death of unknown cause, 1; and acute heart failure, 1.The 3-year progression-free survival rate was 71.2% (Figure 1b). Of the 48 CR patients, recurrences were detected in 9 patients from 8 to 41 months after CRT initiation. The local recurrences of 2 patients were treated completely with ER. Lymph node recurrences were found in 7 patients who underwent salvage surgery or chemotherapy. Of the limited 48 CR patients, the 3-year progression-free survival rate was 81.5%.

**Table 2:** **Characteristics patients experiencing treatment failure after CRT**

No.	Tumor location	Response	Time to failure	Survival	Recurrent location	Treatment after failure
1	Lt	CR	41	Deceased	LN	Chemotherapy
2	Mt	CR	30	Deceased	LN	Surgery
3	Lt	CR	30	Survive	Local	ESD
4	Mt	CR	28	Deceased	LN	Chemotherapy
5	Lt	CR	20	Survive	LN	Surgery
6	Mt	CR	20	Survive	LN	Surgery
7	Mt	CR	10	Deceased	LN	Chemotherapy
8	Mt	CR	8	Survive	Local	ESD
9	Ut	CR	8	Deceased	LN	Surgery
10	Mt	No CR	8	Deceased	Liver	Chemotherapy
11	Mt	No CR	5	Survive	LN	Surgery
12	Mt	No CR	4	Survive	Local	Surgery
13	Mt	No CR	4	Survive	Local	Surgery
14	Mt	No CR	4	Deceased	LN	Surgery
15	Mt	No CR	3	Unknown	Lung	Chemotherapy

UT, upper thoracic; MT, middle thoracic; LT, lower thoracic; LN, lymph node; ESD, endoscopic submucosal dissection.

### Lymph node enlargement

We performed a scheduled thoraco-abdominal CT before and after the treatment according to the protocol for detecting metastatic recurrence. Although no enlarged lymph node was detected by CT before CRT, the enlarged lymph nodes were detected in 20 (36.3%) of the 55 patients during the scheduled follow-up (Table 3). Initially, the salvage surgery including lymphadenectomy for lymph node enlargements were performed in 4 patients. Two patients who underwent surgery were pathologically diagnosed with benign enlarged lymph nodes. Then, we monitored patients with newly enlarged lymph nodes using CT to determine whether the nodes were metastatic recurrences. Among the 20 patients in whom enlarged lymph nodes were detected after CRT, 7 patients were diagnosed with lymph node recurrences because of the additional growth of lymph nodes within 6 months or surgical pathological examinations. The size of the lymph nodes of 13 patients did not change within 6 months, and they satisfied the “benign” definition. However, in 2 patients, the lymph nodes (patients 3 and 6) grew slowly and were finally diagnosed as metastatic recurrences more than 1 year after their initial detections. Consequently, 9 of these 20 patients (45%) were diagnosed with metastasis, and 11 patients (55%) were considered to have reactive enlargement of lymph nodes. The average interval from initiating CRT to the point of lymph node enlargement was 14.1 months in the patients with recurrent metastasis and 12.0 months in the patients with benign lymph node enlargement. The average lymph node diameters were 9.3 mm in the patients with recurrent metastasis and 8.3 mm in the patients with benign lymph node enlargement. We did not find significant differences between the recurrence and the benign enlargements in the interval after CRT or lymph node size. The benign lymph node enlargements that were diagnosed were followed for >19 months, except for 1 patient who was followed for 8 months, which suggests that these patients had benign lymph node enlargements. Regarding the benign lymph node enlargements, FDG accumulation on PET-CT was positive in one patient and negative in 5 patients, and 5 patients did not undergo PET-CT. All of enlarged lymph nodes diagnosed as metastatic recurrence finally showed FDG accumulation. Regarding the alteration of lymph node diameter after detecting lymph node enlargement, we observed that several nodes shrank promptly, whereas others maintained the same size during the follow-up period. Half of the lymph node enlargements that occurred after CRT were eventually diagnosed as false-positive, which means that they could avoid unnecessary treatment.

**Table 3:** **Characteristics of lymph node enlargement**

Metastasis										
No.	Tumor location	Efficacy	LN location	LN size	Time to LN enlargement	LN size after follow-up	Follow-up until final diagnosis	FDG accumulation	Pathology	Salvage treatment
1	Mt	CR	Mediastinum	21 mm	17 months	35 mm	3 months	Positive	SCC	Surgery
2	Mt	CR	Supraclavicular	12 mm	19 months	20 mm	1 month	Positive	SCC	Surgery
3	Lt	CR	Recurrent nerve/supraclavicular	8 mm	27 months	12 mm	14 months	Positive	-	Chemotherapy
4	Mt	CR	Cardia	8 mm	5 months	11 mm	5 months	Positive	-	Chemotherapy
5	Mt	CR	Cervical paraesophageal	7 mm	30 months	7 mm	-	Positive	SCC	Surgery
6	Mt	CR	Mediastinum/supraclavicular	6 mm	14 months	12 mm	14 months	Positive	-	Chemotherapy
7	Ut	CR	Recurrent nerve	6 mm	8 months	10 mm	2 months	Positive	SCC	Surgery
8	Mt	No CR	Left gastric artery	10 mm	5 months	10 mm	-	Positive	SCC	Surgery
9	Mt	No CR	Supraclavicular	6 mm	2 months	10 mm	2 months	Positive	SCC	Surgery
**Benign enlargement**										
**No.**	**Tumor location**	**Efficacy**	**LN location**	**LN size**	**FDG accumulation**	**Time to LN enlargement**	**Duration until LN shrinkage**		**Pathology**	**Follow-up after LN enlargement**
1	Lt	CR	Infradiaphragmatic	19 mm	Negative	2 months	-		-	59 months
2	Lt	CR	Main bronchus	17 mm	Negative	9 months	3 months		-	64 months
3	Mt	CR	Mediastinum	10 mm	Negative	19 months	-		-	22 months
4	Mt	CR	Recurrent nerve	10 mm	Positive	16 months	Up to surgery		Benign	46 months
5	Mt	CR	Subcarinal	10 mm	N.A.	29 months	-		-	19 months
6	Mt	CR	Supraclavicular	10 mm	Negative	2 months	12 months		-	60 months
7	Mt	CR	Superficial cervical	9 mm	N.A.	39 months	-		-	8 months
8	Mt	CR	Recurrent nerve	9 mm	N.A.	5 months	Up to surgery		Benign	78 months
9	Ut	CR	Main bronchus	8 mm	N.A.	2 months	29 months		-	68 months
10	Ut	CR	Cardia	6 mm	Negative	4 months	7 months		-	51 months
11	Mt	CR	Main bronchus	6 mm	N.A.	3 months	-		-	33 months

UT, upper thoracic; MT, middle thoracic; LT, lower thoracic; LN, lymph node; SCC, squamous cell carcinoma; N.A., not assessed.

## Discussion

The current study demonstrated favourable therapeutic outcomes, including CR rate, overall survival, and progression-free survival, as previously reported, for the ESCC patients treated with CRT [10]. More importantly, the present study clearly demonstrated the high incidence of lymph node enlargements found during the follow-up after CRT.

Salvage surgery was performed in the early cases as soon as we detected an enlarged lymph node by CT during the follow-up after CRT. However, we noticed that in the pathological examination after surgery, benign lymph node enlargements occasionally occurred early in several cases. For the later cases, we closely followed lymph node enlargement after CRT to determine the likelihood of a recurrence or of a benign enlargement clinically. The present study indicated that the benign enlargements accounted for >50% of all enlarged lymph nodes detected by CT scan. Based on the premise that benign lymph node enlargements are frequently detected after CRT, we must distinguish recurrences from simple enlargements using non-invasive modality. In the present study, we defined the lymph node enlargement as “benign” when the exacerbation was not detected for >6 months. However, 2 patients finally suffered from metastatic lymph nodes, although their enlarged lymph nodes were not exacerbated within 6 months after initiating CRT. Presently, to avoid unnecessary salvage esophagectomy and lymphadenectomy, we must closely monitor patients with enlarged lymph nodes. Additional studies regarding modalities, biomarkers, or clinicopathological characteristics that discriminated between lymph node enlargement and recurrence should be performed.

With regard to feasibility, adverse events were tolerable compared to the previous study [10]. However, we must recognise the treatment risk after CRT, particularly cardiac toxicity. One patient died within 3 months of initiating treatment. Three patients suffered from cardiac disease accompanied by pericardial effusion as adverse events. We are uncertain whether CRT was directly related to the cardiac disease observed in these cases, because 2 of the 3 patients had been previously treated for myocardial infarction or angina. Moreover, although pericardial effusion was not induced, acute heart failure occurred in 1 patient who had been previously treated for angina, and 2 patients died suddenly from unknown causes. In all cases, the primary tumors were located in the inferior esophagus. The pericardial exposure with radiation might have caused the cardiovascular events, such as heart failure or arrhythmia. These results suggest that we must pay particular attention to those patients who have previous histories of cardiac diseases and inferior esophageal tumors.

The present study has several limitations. First, this is a retrospective study in a single hospital, and the total patient population is small. Second, the mechanism of benign enlargements of lymph nodes is uncertain. It is possible that the decrease of radiation dose contributes the suppression of lymph node enlargements. However, in the present study, lymph node enlargements did not depend on whether they included within or out of radiation field. Third, the histological type of all patients enrolled in this study was squamous cell carcinoma. It is uncertain whether these findings shown in this manuscript are applicable to esophageal adenocarcinoma which is common in Western countries.

## Conclusion

In conclusion, our study reveals that CRT is effective and feasible for patients with clinical stage I ESCC and that lymph node enlargements are often detected during follow-up after CRT. Note that benign lymph node enlargements comprise >50% of the enlarged lymph nodes after CRT.

## Abbreviations

CR: Complete response
CRT: Chemoradiotherapy
CT: Computed tomography
ER: Endoscopic resection
ESCC: Esophageal squamous cell carcinoma
NBI: Narrow band imaging.

## References

[1] Parkin DM, Bray F, Ferlay J, Pisani P (2005). Global cancer statistics, 2002. CA Cancer J Clin.

[2] Muro K (2008). A phase II study of chemoradiotherapy in patients with stage II, III esophageal squamous cell carcinoma (ESCC): (JCOG 9906). J Clin Oncol.

[3] Kato H, Tachimori Y, Mizobuchi S, Igaki H, Ochiai A (1993). Cervical, mediastinal, and abdominal lymph node dissection (three-field dissection) for superficial carcinoma of the thoracic esophagus. Cancer.

[4] Ando N, Iizuka T, Ide H, Ishida K, Shinoda M, Nishimaki T, Takiyama W, Watanabe H, Isono K, Aoyama N, Makuuchi H, Tanaka O, Yamana H, Ikeuchi S, Kabuto T, Nagai K, Shimada Y, Kinjo Y, Fukuda H (2003). Surgery plus chemotherapy compared with surgery alone for localized squamous cell carcinoma of the thoracic esophagus: a Japan Clinical Oncology Group Study–JCOG9204. J Clin Oncol.

[5] Ando N, Kato H, Igaki H, Shinoda M, Ozawa S, Shimizu H, Nakamura T, Yabusaki H, Aoyama N, Kurita A, Ikeda K, Kanda T, Tsujinaka T, Nakamura K, Fukuda H (2012). A randomized trial comparing postoperative adjuvant chemotherapy with cisplatin and 5-fluorouracil versus preoperative chemotherapy for localized advanced squamous cell carcinoma of the thoracic esophagus (JCOG9907). Ann Surg Oncol.

[6] Herskovic A, Martz K, al-Sarraf M, Leichman L, Brindle J, Vaitkevicius V, Cooper J, Byhardt R, Davis L, Emami B (1992). Combined chemotherapy and radiotherapy compared with radiotherapy alone in patients with cancer of the esophagus. N Engl J Med.

[7] Cooper JS, Guo MD, Herskovic A, Macdonald JS, Martenson JA, Al-Sarraf M, Byhardt R, Russell AH, Beitler JJ, Spencer S, Asbell SO, Graham MV, Leichman LL (1999). Chemoradiotherapy of locally advanced esophageal cancer: long-term follow-up of a prospective randomized trial (RTOG 85–01). Radiation Therapy Oncology Group. JAMA.

[8] Coia LR, Engstrom PF, Paul AR, Stafford PM, Hanks GE (1991). Long-term results of infusional 5-FU, mitomycin-C and radiation as primary management of esophageal carcinoma. Int J Radiat Oncol Biol Phys.

[9] Ohtsu A, Boku N, Muro K, Chin K, Muto M, Yoshida S, Satake M, Ishikura S, Ogino T, Miyata Y, Seki S, Kaneko K, Nakamura A (1999). Definitive chemoradiotherapy for T4 and/or M1 lymph node squamous cell carcinoma of the esophagus. J Clin Oncol.

[10] Kato H, Sato A, Fukuda H, Kagami Y, Udagawa H, Togo A, Ando N, Tanaka O, Shinoda M, Yamana H, Ishikura S (2009). A phase II trial of chemoradiotherapy for stage I esophageal squamous cell carcinoma: Japan clinical oncology group study (JCOG9708). Jpn J Clin Oncol.

[11] Smith TJ, Ryan LM, Douglass HO, Haller DG, Dayal Y, Kirkwood J, Tormey DC, Schutt AJ, Hinson J, Sischy B (1998). Combined chemoradiotherapy vs. radiotherapy alone for early stage squamous cell carcinoma of the esophagus: a study of the Eastern Cooperative Oncology Group. Int J Radiat Oncol Biol Phys.

[12] Yamamoto S, Ishihara R, Motoori M, Kawaguchi Y, Uedo N, Takeuchi Y, Higashino K, Yano M, Nakamura S, Iishi H (2011). Comparison between definitive chemoradiotherapy and esophagectomy in patients with clinical stage I esophageal squamous cell carcinoma. Am J Gastroenterol.

[CR13] The pre-publication history for this paper can be accessed here:http://www.biomedcentral.com/1471-2407/14/706/prepub

